# The effects of reminiscence therapy on depressive symptoms of Chinese elderly: study protocol of a randomized controlled trial

**DOI:** 10.1186/1471-244X-12-189

**Published:** 2012-11-05

**Authors:** Ting-ji Chen, Hui-jie Li, Juan Li

**Affiliations:** 1 Center on Aging Psychology, Key Laboratory of Mental Health, Institute of Psychology, Chinese Academy of Sciences, Beijing, China

## Abstract

**Background:**

Depression is one of the most common mental disorders with a high prevalence among the older adults. In recent years, after realizing some side effects of the antidepressants, non-pharmacological psychological treatments begin to attract accruing attention. Reminiscence therapy is one of the psychological treatments that specially designed for the elderly to improve their mental health status by recalling and assessing their existing memory. Though some studies indicate reminiscence therapy can be effective and beneficial for the mental health of elderly, the conclusions are not consistent yet. The aim of this research is to assess the effectiveness of reminiscence therapy for Chinese elderly.

**Methods:**

Sixty older adults (≥60 years of age) with mild to moderate depression will be randomly assigned to an experimental or a control condition. The participants in the experiment group will receive the reminiscence therapy under the Watt’s protocol with adaptation to Chinese Culture which consists of six weekly sessions of 90 minutes each. The control group will be treated as before. An assessor who is blind to intervention will conduct the measures before treatment, after treatment immediately, and three months after treatment.

**Discussion:**

This study will provide the evidence whether the reminiscence therapy is effective to treat depressive symptoms of Chinese elderly. This research has been registered in the clinicaltrials.gov (NCT01553669).

## Background

Aging population around the world is growing fast. As the United Nations Statistics Division shows, the elderly of the world was about 865 million in 2010, and will reach 2.008 billion in 2050 [1]. Moreover, according to National Bureau of Statistics of China, the Chinese elderly aged 60 and above were about 178 million in 2010, accounting for 13.26% of population [2]. With the increasing number and proportion of the elderly, the geriatric mental health problem becomes more important. Depression is one of the most common mental disorders found in the elderly. In our recent survey of Chinese elderly, the prevalence of self-reported depressive symptoms (CES-D≥16) reached to 39.86% [3]. Depression causes disability of functional impairment, reduces life satisfaction and quality, it is also associated with a high risk of suicidal behavior, and increases the burden and the costs of health and home care services [4-6]. Therefore, the appropriate early intervention for the elderly individual suffering depression is significant and critical [7,8].

At present, drug therapy and psychotherapy are two commonly used methods for the treatment of depression [9]. Depression is mainly treated by medication for a long time [10]. However, previous studies also indicated that pharmacologic approaches had numerous side effects [7,11-13]. In addition, the elderly cannot learn any skills from the drug therapy in protection against the relapse of depression [8]. Currently, increasing studies and reviews support the efficiency of psychotherapeutic treatment for depression. Cognitive or cognitive behavioral therapy [14-16], problem-solving therapy [17] and interpersonal therapy [18,19] are widely accepted as methods in reducing the depression. Based on traditional interventions, reminiscence therapy is specially recommended for geriatric depressive adults. This psychotherapy has been designed and developed because of the recognition of the unique needs and concerns involved in adaptation to the late stage of life [8].

### Reminiscence therapy

The theoretical framework of reminiscence therapy derived from Erikson’s theory of ego development [20]. Erikson divided life span into eight stages. In his theory, Erikson believed people would experience a main crisis or conflict in each stage, which served as a turning point in development and needed to be solved. For the elderly, the principle task is to achieve ego-integrity and avoid despair [21]. Erikson suggested that assessment of the past was an essential component in successful completion of this task [8]. Butler and Birren developed Erikson’s work and proposed that ego integrity could be achieved in an analytical and evaluative way to recall one’s past [22]. In recent years, researchers developed the continuity theory and contributed other insights in understanding the importance of reminiscence. According to continuity theory, when individuals encounter life-events which need them to change or convert, they will use adaptive strategy by linking present to their past experiences. This strategy is adopted to “preserve and maintain existing internal and external structures” and “produce continuity in inner psychological characteristics as well as in social behavior and in social circumstances” [23].

Reminiscence therapy is a method of using the memory to protect mental health and improve the quality of life. Reminiscence is not just to recall the past events or experiences. It is a structured process of systematically reflecting on one’s life with a focus on re-evaluation, resolving conflicts from the past, finding meaning in one’s life and assessing former adaptive coping responses [24-26]. Watt and Wong (1991) defined six types of reminiscence: integrative, instrumental, transmissive, narrative, escapist and obsessive [27]. They identified the integrative and instrumental reminiscence was related to adaptation and well-being among older adults, and developed the corresponding intervention manual [25].

In the last several decades, increasing studies indicated that reminiscence therapy is effective for the elderly with depression [26,28-33]. Besides alleviating the depression symptoms, reminiscence therapy was proved to improve self-esteem [34], life satisfaction [35], psychological well-being [36], personal mastery [37,38] and loneliness [39,40]. However, the conclusion is not consistent [41-43]. The reasons may be attributed as some studies did not follow randomized controlled trial (RCT) design, lack of strict protocol and had high dropout rates.

### Study purposes

The purpose of the research is to examine the effect of reminiscence therapy on depressive symptom, self-esteem, life satisfaction, and loneliness in Chinese elderly.

Specifically, the aims are:

Firstly, we intend to explore whether reminiscence therapy will significant improve the experiment group’s depressive symptom in comparison with control group after six weeks therapy, and whether the efficacy can be maintained during three-month follow-up.

Secondly, we are interested in whether reminiscence therapy is a potential effective treatment to reduce the loneliness, increase the self-esteem and life satisfaction for the experiment group elderly.

## Method

### Design

The trial will be conducted as a pre-post test compared and randomized controlled trial. After collection of the baseline test, research assistant will open a sealed sequential randomization envelope for each participant indicating either the “reminiscence therapy group” or the “waiting-list control group”. The envelopes have been prepared previously by the evaluator via a computerized randomized program. The assessments will be conducted by another evaluator who is not the therapist and is blind to the group assignment.

**Figure 1:**
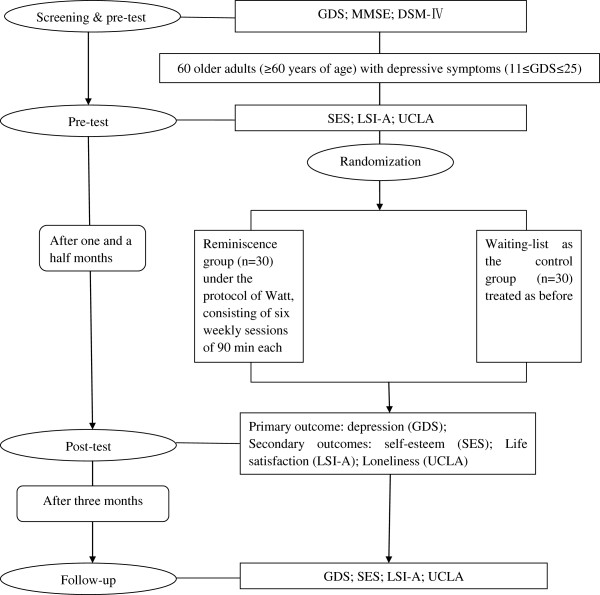
**An overview of the study design. **Abbreviations: GDS =Geriatric Depression Scale; MMSE = Mini-Mental State Examination;DSM-IV = Diagnostic and Statistical Manual of Mental Disorders, Fourth Edition; SES = Self-Esteem Scale; LSI-A = Life Satisfaction Index A; UCLA = Russell’s UCLA Loneliness Scale.

The reminiscence therapy group participants will receive six-week interventions, while the control group participants will not receive any emotional related treatment. After the intervention, all the participants will receive post-test. Then after three months, all the participants will receive the follow-up test. An overview of the study design is shown in Figure 1.

The research protocol has been approved by the Institutional Review Board of the Institute of Psychology, Chinese Academy of Sciences. Before signing a consent form to get into the research, the participants will be introduced about the procedure, the purpose and the objectives, the items we will use and the time needed of this study. They will be informed that they are free to withdraw at any time and we promise their personal information will never be released.

### Participants

A total of sixty subjects who fulfill the following inclusion and exclusion criteria will be recruited from the community.

#### Inclusion criteria

1) The age of participants is equal or over than 60 years old;

2) The score of the Geriatric Depression Scale (GDS) should fall between 10 and 26 points;

3) A score of 24 or higher on the Mini-Mental State Examination (MMSE);

4) Not taking anti-depressant medication or taking part in other psychotherapy when enrolling, or with stable anti-depressant medication for at least three months;

5) Having no problem with communication (such as, can speak fluent mandarin, or, his/her dialect can be understood by other participants or evaluators).

#### Exclusion criteria

1) Suicide attempt;

2) A diagnosis of cognitive impairment by DSM-IV, or mental disorders other than depression;

3) Alcohol or drug abuse;

4) Cannot grantee attending the therapy at a weekly base due to physical impairment, lack of time or unwilling etc.

### Intervention

#### Experiment group

The subjects who are arranged to the experiment group will be intervened using the reminiscence therapy manual proposed by Watt and Cappeliez (2000). According to the manual, the group consisted of six weekly sessions of 90 min each. There will be about four people in each subgroup. During each weekly session, the memories recalled focused on a different theme. The themes are as follows: major branching points in your life, family life, your career or major life work, your loves and hates, stress experiences, the meaning of your life and belief. Five relative questions were set to help retrieve memory in each theme. We explain to participants that the memories of these themes should be related to experiences with a significant impact on their life. Also, we make a distinction between personal recollections and recollections of historical facts which are not the focus of the intervention [25].

Except for the first session, the other sessions share the same procedures, including: homework review and agenda development (10 minutes), relaxation and focusing on reminiscence (5 minutes), contact work (50 minutes), feedback to clients (15 minutes), assignment of homework (5 minutes), feedback to therapist and questions (5 minutes). The first session will begin with participants’ self-introductions to get to know each other. The therapist will offer information about his work and qualifications to get confidence and closer to the participants. Then he will explain some concepts used in the session (such as “round”), rationale for reminiscence therapy, the preparations of the each intervention and corresponding procedures.

During each therapy process, Contact Work will account for a large proportion. Contact Work is a one-to-one encounter between a single therapist and a client in the group setting [25]. The therapist will invite the participant to share his/her memory about the theme, which he/she is asked to prepare before the meeting. With the direction of the manual, reminiscence will be conducted structurally. The other group members are required to listen and give no comment or judgment unless therapist’s request at that moment. As another necessary element, homework is very important for the reminiscence therapy. Homework which is related to the theme of the next session is assigned at the end of the present session and will be reviewed at the beginning of the next session. There are several reasons for stressing these homework assignments. For the first, it can help the participants re-familiarize themselves with the memory and the context in which it occurred, makes them get involved into the therapy quickly, and uses the time of the session more efficiently. Secondly, we can use homework to assess the compliance of the participants. Lastly, it can facilitate participants to practice this skill.

#### Control group

The participants assigned to the waiting-list as control group will be treated as before. We will keep in touch with them getting to know whether they are still available to be the member of the control group.

### Data collection and outcome measures

The main data will be collected using some standardized instruments with demonstrated validity and reliability which will be described as follows. Data collection will occur on screening (as for baseline or pre-test), after intervention immediately (as for post-test), and three months after the intervention (as for follow-up). In addition to these measures, we will use some scales developed by ourselves to collect data. These scales are used to assess the participants’ attitude towards the intervention. Moreover, the therapist will be given the scales used to rate participants’ compliance after each session.

#### Primary outcome: Depressive symptoms

Symptoms of depression will be measured with the GDS. GDS is a self-rated scale consisting of 30 items with response of “yes” or “no” to measure a person’s emotional state in the past week. The entire scores ranges from 0 to 30, and higher scores represent more severe level of depression. Generally, scores from 11 to 20, from 21 to 30 indicate mild, severe depression respectively [44]. The GDS designed specifically for the elderly, and it has been proved with high reliability and validity. According to the research of Yesavage and Brink (1982), the Cronbach’s alpha coefficient was 0.94 and the test-retest reliability after one week was 0.85 among twenty participants [45].

#### Secondary outcomes: self-esteem, life satisfaction, and loneliness

Self-esteem will be measured using the Rosenberg self-esteem scale (SES). The SES consists of 10 items, with responses from 1 (completely agree) to 4 (completely disagree). The total score ranges from 10 to 40, higher scores indicating higher self-esteem. It was found to have a high reliability and validity [46]. For the 8th item (I wish I could have more respect for myself), in consideration of cultural difference, some studies suggest and prove that the way of expression should be changed to negative form when it is translated into Chinese [47,48].

Life satisfaction will be measured with the Life Satisfaction Index A (LSI-A), which has been proved having high reliability and validity [49]. LSI-A is consisted of 20 items for which an “agree” or “disagree” or “not sure” response is required. The total score ranges from 0 to 20, and higher total score indicates better life satisfaction.

Loneliness will be measured using the third edition of Russell’s UCLA Loneliness Scale which consists of 20 items, with responses from 1 (never) to 4 (often) [50]. The total score ranges from 20 to 80, with higher score indicating a higher sense of loneliness. According to the research of Russell (1996), the alpha coefficient was 0.89 and the test-retest reliability after twelve months was 0.73 among the elderly [51].

### Statistical analysis

According to some previous researches, personal characteristics of the subjects may have an impact on the outcomes. Though the RCT design is adopted, the demographic data between the control group and the experiment group still should be considered. When the two groups do not match on demographic data, related statistical methods will be brought in to make up for this. In addition, the data of participants who are absent from the group session for four times or more and who turn to antidepressant or other non-pharmaceutical intervention before the end of the therapy will be excluded when analyses conducted [39].

SPSS 16.0 statistical packages will be used to conduct the data analyses. Paired *t* test will be used to detect the changes of the outcome measures within intervention or control group. Standardized change scores will be calculated by subtracting pre-test score from post-test score of intervention and dividing by the SD for all participants combined. A repeated measures analysis of covariance (ANCOVA) model will be used to evaluate the effects of intervention on continuous outcome variables that are available at each data collection point. The between-subjects factor is group (intervention group, control group) and the within-subjects factor is assessment phase (baseline, post-test, follow-up test). All statistical tests were two sided, and *p* values < 0.05 were considered significant. In addition, effect sizes (Cohen’s d) [52] will be computed based on the differences between the pre-and post-intervention (or follow-up test) means and the pooled variance.

## Discussion

Reminiscence is used as a psychological therapy for depression especially beneficial for elderly adults for the following reasons. First, the reminiscence does not ask for some new complicated skills for elderly, it just employs the resources of them. Therefore it is very suitable for the elderly who decline in cognitive and other functions [8,20]. Secondly, the participants are familiar with their memories. They are the main actors in their stories. It makes them feel comfortable, provides a sense of control and gets them involved in the therapeutic process quickly. Furthermore, it is easier to handle for the therapist than other kinds of intervention, such as cognitive therapy. Though depending on the clinical experience, it still can be used by the graduate student in psychological or nurse who has been trained [41]. Finally, it has been found harmless so far [7].

We also examine whether participants’ attitude and anticipation of the intervention will impact the effect of the intervention. In a research conducted by Arean et al. (1993), participants were requested to anonymously complete a questionnaire about their expectation of the treatment and therapist competency. The finding indicated that subject's perceptions of treatment efficacy, or therapist competency did not influence the outcome measures [28]. However, Bohlmeijer et al. (2009) proposed people who had more positive and general attitude towards reminiscence therapy would benefit more than people who were less interested in that [37]. We will examine these factors in the study. In addition, we will examine the level of participants’ engagement from the therapist’s view to find out whether it will impact the effect of the therapy. This has not been studied in previous studies.

The aim of this study is to evaluate the effects of reminiscence therapy on depression, self-esteem, life satisfaction and loneliness of Chinese elderly. Since the cultures, the values, and the way for expressing of Chinese elderly are different from elderly westerners, it is necessary to verify the applicability of treatment effect of reminiscence into Chinese elderly. Two studies have been conducted in mainland of China [53,54]. However, some methodological shortages limit generalization of the results, such as not RCT design, no well-described protocol and follow-up measurements, which are defined as core characteristics of high quality studies [41]. We will conduct our study following these standards and more strict recruiting criteria to examine whether the reminiscence therapy is effective for Chinese elderly. If the findings of this research are proved to be positive, then we could say it may provide an alternative non-pharmacological therapy that is effective to reduce the depressive symptoms and improve the living quality of potential large older populations in China.

## Competing interests

The authors declare that they have no competing interests.

## Authors’ contributions

JL conceived the trial. TJC designed the detailed protocol. TJC and HJL wrote the first draft of the paper. All authors contributed to development and revision of the manuscript and take public responsibility for its content. All authors have read and approved the final manuscript.

## Pre-publication history

The pre-publication history for this paper can be accessed here:

http://www.biomedcentral.com/1471-244X/12/189/prepub
